# Comparative Evaluation of Remaining Dentin Thickness Using Manual Protapers, Protaper Gold, and Reciprocating Waveone Gold in Single-Rooted Teeth With Cone-Beam Computed Tomography: An In-Vitro Study

**DOI:** 10.7759/cureus.76610

**Published:** 2024-12-30

**Authors:** Simran Utwal, Manu Bansal, Aabhash A Agarwal, Gargee Karmveer, Prishita Malani, Rashika Jauhari, Diksha Verma

**Affiliations:** 1 Conservative Dentistry and Endodontics, Jaipur Dental College, Jaipur, IND; 2 Pedodontics, Jaipur Dental College, Jaipur, IND; 3 Prosthodontics, Jaipur Dental College, Jaipur, IND

**Keywords:** cone-beam computed tomography (cbct), protaper gold file, protaper universal, reciprocating motion, remaining dentine thickness (rdt), waveone gold

## Abstract

Aim: The aim of the study is to compare the remaining dentinal thickness in the single-rooted tooth at the coronal, middle, and apical third using three different rotary instrumentation techniques using cone-beam computed tomography (CBCT).

Materials and methodology: Sixty-eight noncarious single-rooted teeth were collected and decoronated at the level of cementoenamel junction with a diamond disc. All specimens were randomly divided into three experimental groups - Manual ProTaper Universal (PTU; Dentsply Maillefer, Ballaigues, Switzerland), Protaper Gold (PTG; Dentsply Maillefer), Waveone Gold (Dentsply Maillefer) and one control group of 17 teeth each. After mounting the samples on a modeling wax sheet, preoperative CBCT scans were taken. Biomechanical preparation of canals was done following the assigned protocol of manufacturers. Postoperative CBCT scans were taken, and comparison was carried out with preoperative scans at 2, 5, and 8 mm.

Result: Waveone Gold has the advantage of retaining dentin thickness at all locations but more significantly in the middle and coronal parts when compared to PTG and manual instrumentation (PTU) file systems in single-rooted teeth. PTG also showed promising results in retaining dentin thickness which was comparable to Waveone Gold file system but better than PTU in both buccolingual and mesiodistal direction. PTG also has the advantage of retaining dentin thickness at the apical third in both buccolingual and mesiodistal directions.

Conclusion: Waveone Gold file system performed better and removed lesser dentin in both buccolingual and mesiodistal directions. More dentin was removed at the coronal and middle in the mesiodistal direction with the use of PTU and PTG, and a significant difference was seen between the Waveone Gold group and other groups in the study.

## Introduction

The three main factors contributing to endodontic treatment outcomes are three-dimensional obturation, disinfection, and canal preparation. The shaping and cleaning of the root canals are very important in performing successful endodontic treatment [1]. Among the most crucial phases of canal therapy is the shaping of the root canal; this phase allows for the evaluation of the success rate of further measures applied in the course of treatment, among which are root canal obturation and chemical disinfection [2].

The root's resistance to fracture is directly impacted by the thickness of dentin that remains following instrumentation [3]. Most of the dentin removal in a root canal is removed during the instrumentation phase. It has been discovered that the mesial and distal directions include the majority of the dentin loss [4].

In order to prepare the tooth for the proper filling, the form also makes it easier to maintain the position and integrity of the apical anatomy and the canal. Conversely, overshaping causes an abnormal reduction in the thickness of the remaining dentin, weakening the root [5]. Because NiTi rotary instrumentation was introduced in the 1980s, root canal shaping has become more homogeneous and predictable, and endodontics are easier and faster compared with manual instrumentation [6].

According to Caputo and Standlee, in order to prevent vertical fracture, 1 mm of tooth structure must surround the post [7]. After preparation, Lim and Stock suggested that the canal walls should have at least 0.3 mm of dentin. This width is sufficient to withstand occlusal stresses and lateral forces during canal obturation [8].

Katz and Tamse showed that the mesial and distal sides of an oval root canal area are the most influenced by intraradicular procedures. Maximum dentin was lost after preparation of the root canal, while less dentin was lost after preparation of the post space, with maximum loss in the mesial and distal directions [9].

Out of all these variables, one of the most crucial ones to take into account for the effectiveness of these treatments is the amount of dentin thickness that remains. The remaining dentin thickness represents the mechanical threshold that one should not cross with the enlargement of the root canal diameter, with the help of these instruments, which can lead to the significant weakness of the dentinal walls [10]. The problem that arises then is to achieve adequate canal taper utilizing any file technique without excessively cutting dentin. Proper selection of the file system is of utmost importance [11].

The utilization of cone-beam computed tomography (CBCT) enhances the sophistication of this study. CBCT has been highly recommended as an extremely promising tool in studying the anatomy of root canals. It utilizes a cone-shaped x-ray beam and a detector, which acquires data in a cylindrical volume in one acquisition. Advantages of CBCT include the creation of highly accurate cross-sectional and 3D images with high resolution; it is fully quantifiable and gives consistent and repeatable results [12].

The purpose of this research was to evaluate the cutting effectiveness of three distinct file systems in relation to the thickness of dentin that remains. Protaper Gold (Dentsply Maillefer, Ballaigues, Switzerland), Waveone Gold (Dentsply Maillefer), and Manual ProTaper Universal (Dentsply Maillefer) were the file systems utilized in this investigation.

## Materials and methods

Study design and ethical considerations

This study was designed as a clinical study to evaluate the remaining dentin thickness using different rotary systems in single-rooted teeth. After receiving the Institutional Ethical Committee clearance certificate, MVGU/ADM/2022/144 from Maharaj Vinayak Global University, Jaipur Dental College, Jaipur, India, an in vitro study was carried out in the Department of Conservative Dentistry and Endodontics.

Sample size calculation

The sample size has been estimated using the software GPower v. 3.1.9.2 (G-Power, Aichach, Bavaria). Considering the significance level of 0.05, the power of the study is at 80% and the margin of error is 5%, the total sample size needed is 68. Hence, the sample size comprises 17 samples per group.

Sample preparation 

A total of 68 noncarious, removed human single-rooted teeth were obtained. The specimens were gathered from the Department of Oral and Maxillofacial Surgery at Jaipur Dental College, Jaipur, Rajasthan, India, ensuring a homogenous sample that minimized variability due to anatomical differences. The research includes teeth extracted without any internal or else external problematic root resorption as well as with apical closure present. Teeth exhibiting pathological root resorption, major root angulation, as well as immaturity were excluded from the study. Selected teeth for the study were collected and sterilized in 3% NaOCl for 30 minutes as well as then stored in 0.9% normal saline solution. Pre-operative radiographs were taken to confirm the patency of the canal.

After that, a diamond disc mounted on a straight handpiece with a slow speed was employed to decoronate the teeth at the level of the CEJ. After that, each group of 17 specimens was randomly assigned to one of three experimental groups and one control group. Group A - Manual Protaper Universal, Group B - Protaper Gold, Group C - WaveOne Gold, and Group D - Control.

**Figure 1:**
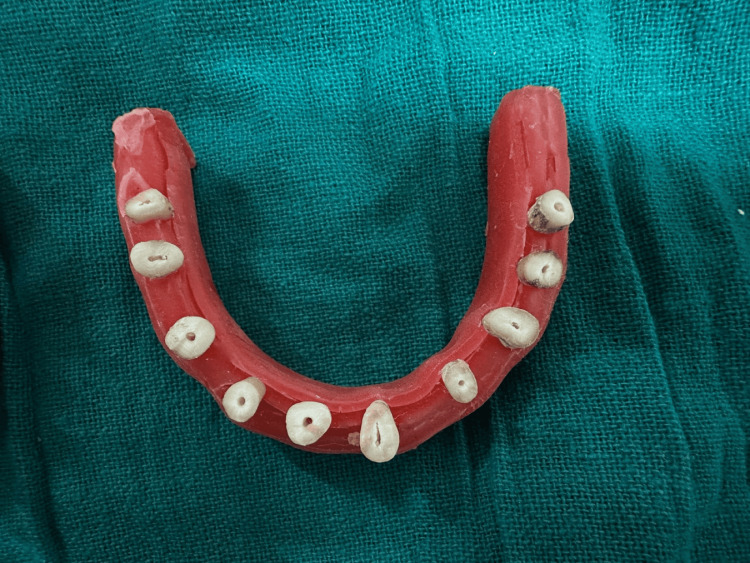
Sample mounting on modeling wax

**Figure 2:**
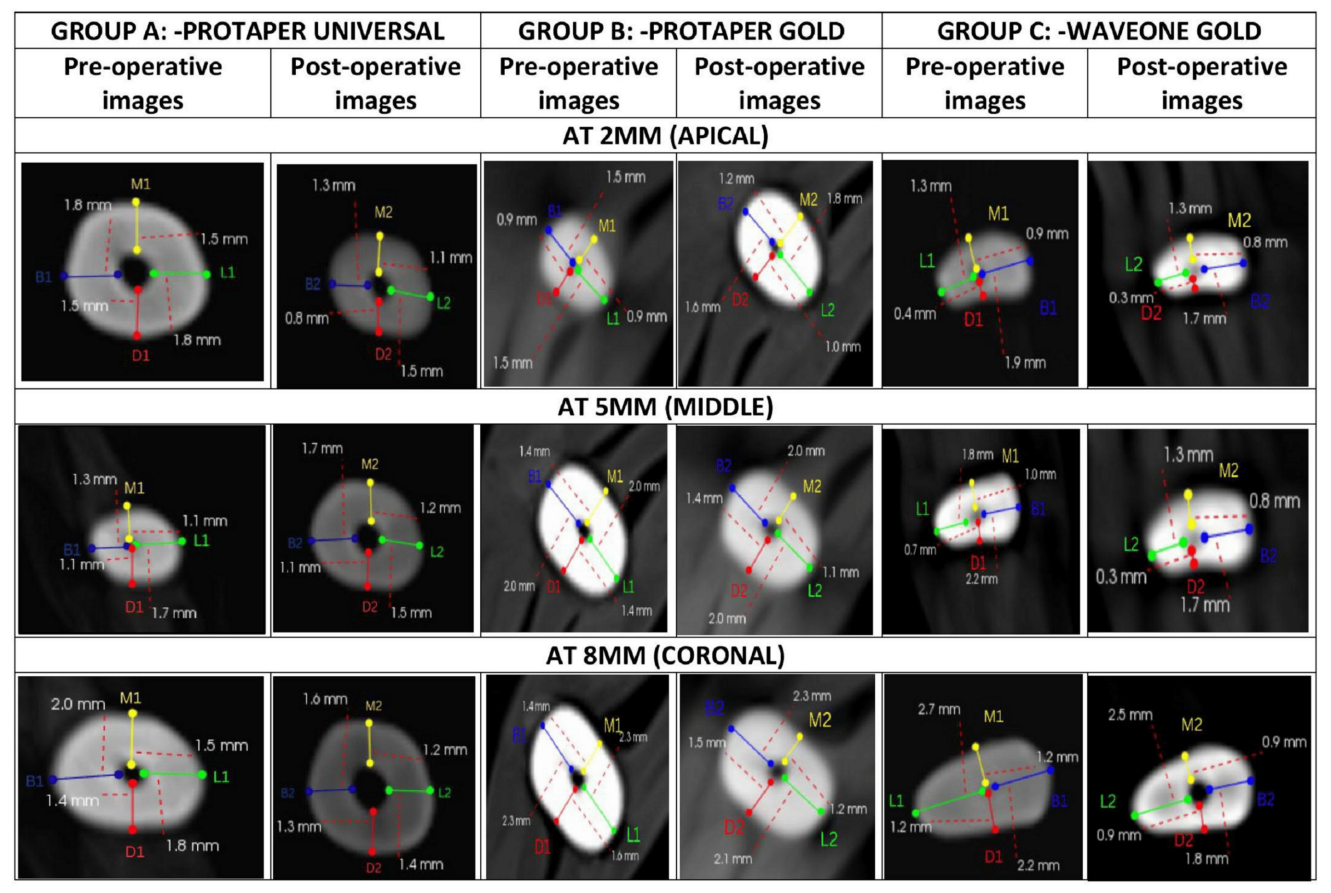
Pre and post-operative images of tooth depicting remaining dentin thickness using cone-beam computed tomography

A no. 10K file (Mani Inc., Japan) was utilized to create a glide path and a no. 15K file was utilized to measure the canal's length and subtract 0.5 mm from the result to get the working length. The samples from every group were subsequently placed on a modeling wax sheet (Figure 1). A baseline pre-instrumentation CBCT was then obtained for every example. Figure 2 illustrates the utilization of this CBCT to document the residual dentin thickness following biomechanical preparation with different file techniques.

Group A (Protaper Universal)

The no. 10K file in this manual file system investigated the canal first, followed by the no. 15K file, which did the same while winding a watch. SX(orange), S1(purple), S2(white), F1(yellow), as well as F2(red) are the basic sequences utilized in Hand Protaper files (n=17). With a crown-down method, every root canal was prepared by the technology of Protapers. The biomechanical sample preparation process took roughly five to six minutes.

Group B (Protaper Gold)

The basic sequence utilized in Protaper files is - SX(orange), S1(purple), S2(white), F1(yellow), as well as F2(red). The file was used in an X-Smart Plus endomotor (Dentsply Maillefer) in a continuous clockwise rotational motion at a speed and torque for Sx and S1-300 rpm and 5.10 torque, for S2 and F1-300 rpm and 1.50 torque and for F2-300 rpm and 3.10 torque as suggested by the manufacturers.

Group C (WaveOne Gold)

The primary red 21 mm is available in ISO 25 tip and 7% taper for most canals; small yellow 21 mm is available in ISO 20 tip and 7% taper for small canals; medium is available in ISO 35 tip and 6% taper; large black 25 mm is available in ISO 45 as well as 5% taper for large canals; This reciprocating file system is offered in four distinct single file sizes.

For the biomechanical preparation, the file (n=17) was used in an X-Smart Plus endomotor (Dentsply Maillefer) in reciprocation mode, the endomotor was calibrated by manufacturers, SMALL WaveOne Gold file no. 020.07 as well as PRIMARY WaveOne Gold file no. 025.07 had been utilized. WaveOne Gold rotation is 360° in three cycles (150° counterclockwise and 30° clockwise directions) with a speed of 350 rpm. The range of movement of the file was 3 mm. After every three successive movements, the file was taken out, cleaned, and washed with distilled water until the full working length was reached. It was performed by a single person (postgraduate student from the Department of Endodontics) under the guidance of an Endodontist with 15 years' experience.

Group D (Control)

The teeth (n=17) were left unprepared. Between each file, irrigation was done with normal saline and 2mL 3% sodium hypochlorite (Hyposol, Prevest) and 17%EDTA (RCT prep, Waldent, India). The dentin thickness that remained after the root canal was prepared was measured by comparing the CBCT post-instrumentation scan, which was obtained following biomechanical preparation, with a CBCT pre-instrumentation scan obtained at three different levels: 2, 5, and 8 mm from the apex.

Reliability testing of examinations

Two blinded operators who had received CBCT analysis training (two PhD students from the Department of Oral Medicine and Radiology) performed the measurements. The measurements were examined by an Endodontist with 15 years' experience and a postgraduate student. Both examiners observed the images on the same display. Interexaminer agreement measured with Cohen's kappa between two examiners was calculated to be 0.84 with the percentage of agreement at 84% showing almost perfect agreement.

Analysis of remaining dentin thickness using CBCT

Mean in buccolingual (BL) direction pre-instrumentation: $Mean BL_{1}=\frac{B_{1}+L_{1}}{2}$

Mean in Mesiodistal direction (MD) pre-instrumentation: $Mean MD_{1}=\frac{M_{1}+D_{1}}{2}$

Mean in BL direction post-instrumentation: $Mean BL_{2}=\frac{B_{2}+L_{2}}{2}$

Mean in MD post-instrumentation: $Mean MD_{2}=\frac{M_{2}+D_{2}}{2}$

Removed dentin in BL direction $(BL)=(BL_{1}-BL_{2})$ Removed dentin in MD: $(MD)=(MD_{1}-MD_{2})$

Statistical analysis

The data were entered into Microsoft Excel and analyzed using SPSS (Statistical Package for Social Sciences) package 26.0 (IBM Corp., Armonk, NY) for relevant statistical comparisons. Results were presented in the form of tables and graphs. Shapiro Wilk test was used to check whether the continuous variables were following normal distribution or not. As data were normally distributed one-way ANOVA test was used for the comparison of mean values (continuous variables) between more than two groups. Post hoc Tukey test was used for the comparison of values between groups. P values were taken significantly when less than 0.05.

## Results

Table 1 facilitates a meticulous intergroup comparison concerning the mean BL direction measurements at the apical, middle, and coronal thirds of each tooth. The analysis incorporates data from three experimental groups alongside a control group (Group D), which evidently received no endodontic treatment, serving as a baseline for comparison.

**Table 1:** Intergroup comparison for mean buccolingual direction at coronal, middle, and apical thirds of each tooth N - Number of samples, f - Functional value, p - Level of significance, BL - Buccolingual

		N	Mean	Standard deviation	F-value	P-value
					Apical- BL	Group A	17	0.1344	0.11361	15.031	0.000
Group B	17	0.0971	0.07174			
Group C	17	0.1500	0.05303			
Group D	17	0.0000	0.00000			
Total	68	0.0948	0.09134			
Middle BL	Group A	17	0.2656	0.10912	20.886	0.000
	Group B	17	0.1412	0.14387		
	Group C	17	0.1176	0.07058		
	Group D	17	0.0000	0.00000		
	Total	68	0.1291	0.13347		
Coronal- BL	Group A	17	0.3156	0.21270	19.156	0.000
	Group B	17	0.1294	0.08303		
	Group C	17	0.1059	0.09334		
	Group D	17	0.0000	0.00000		
	Total	68	0.1351	0.16400		

In the apical third, statistical analysis revealed significant variations among the groups. Group A exhibited a mean BL direction measurement of 0.1344 (SD = 0.11361), while Group B showed a lower mean of 0.0971 (SD = 0.07174), and Group C demonstrated a mean of 0.1500 (SD = 0.05303), the highest among the experimental groups. The control group (Group D) maintained a mean of 0.0000, indicating no alteration. The F-value stood at 15.031, with a highly significant p-value of 0.000, highlighting significant differences across the groups.

For the middle third, Group A's mean measurement of 0.2656 (SD = 0.10912) was notably higher compared to the other groups, indicating a more substantial reduction in dentinal thickness. Group B and Group C recorded means of 0.1412 (SD = 0.14387) and 0.1176 (SD = 0.07058), respectively. The control group again showed no change, reinforcing its baseline status. The observed F-value was 20.886, with the p-value being 0.000, confirming the statistical significance of the observed differences.

The coronal third analysis presented the most significant disparities. Group A had a mean of 0.3156 (SD = 0.21270), significantly higher than that observed in Group B (mean = 0.1294, SD = 0.08303) and Group C (mean = 0.1059, SD = 0.09334), suggesting a greater impact on the dentinal thickness in this group. The control group, as expected, exhibited no change, with a mean of 0.0000. The F-value was 19.156, with a p-value of 0.000, underscoring the significance of the differences among the groups.

Table 2 presents an intergroup comparison focusing on the mean MD measurements at the apical, middle, and coronal thirds of each tooth, evaluated across four distinct groups, including a control group (Group D). The table showcases the mean values, standard deviations, F-values, and P-values, offering insight into the effects of different endodontic treatments on the mesiodistal dentinal thickness.

**Table 2:** Intergroup comparison for mean Mesiodistal direction at coronal, middle, and apical thirds of each tooth N - Number of Sample, f - Functional Value, p - Level of Significance, MD - Mesiodistal

		N	Mean	Standard deviation	F-value	P-value
					Apical- MD	Group A	17	0.3906	0.14517	58.733	0.00
Group B	17	0.1382	0.07609			
Group C	17	0.1882	0.05736			
Group D	17	0.0000	0.00000			
Total	68	0.1761	0.16292			
Middle-MD	Group A	17	0.3125	0.10408	59.789	
	Group B	17	0.1500	0.06614		
	Group C	17	0.0912	0.06431		
	Group D	17	0.0000	0.00000		
	Total	68	0.1358	0.13195		
Coronal-MD	Group A	17	0.3125	0.11030	17.803	
	Group B	17	0.1618	0.10236		
	Group C	17	0.0824	0.20611		
	Group D	17	0.0000	0.00000		
	Total	68	0.1366	0.16980		

The apical third exhibited significant differences in mean MD measurements among the groups. Group A demonstrated a markedly higher mean of 0.3906 (SD = 0.14517), significantly exceeding the measurements observed in Groups B and C, with means of 0.1382 (SD = 0.07609) and 0.1882 (SD = 0.05736), respectively. The control group (Group D) showed no change, with a mean of 0.0000. The F-value of 58.733 and a P-value of 0.00 indicate these differences are highly significant, suggesting that the treatment corresponding to Group A results in a substantial reduction in dentinal thickness in the apical third compared to other groups.

Similar to the apical third, the middle third also revealed significant intergroup disparities. Group A again recorded the highest mean mesiodistal measurement of 0.3125 (SD = 0.10408). In comparison, Group B had a mean of 0.1500 (SD = 0.06614), and Group C had the lowest at 0.0912 (SD = 0.06431), with the control group maintaining a baseline of 0.0000. With an F-value of 59.789, the statistical analysis confirms significant differences among the groups, highlighting a pronounced effect of the endodontic treatment associated with Group A on the middle third's mesiodistal dentinal thickness.

The coronal third analysis showed Group A with a mean of 0.3125 (SD = 0.11030), significantly higher than both Group B (mean = 0.1618, SD = 0.10236) and Group C (mean = 0.0824, SD = 0.20611), with the control group again showing no alteration. The F-value was 17.803, indicating significant differences across the groups, though with a smaller magnitude compared to the apical and middle thirds. This finding suggests that while the treatment associated with Group A influences the coronal third's dentinal thickness, the impact is somewhat less pronounced than in the other two-thirds.

These findings indicate that the endodontic treatments evaluated, particularly the one associated with Group A, have a significant impact on the mesiodistal dentinal thickness across all thirds of the tooth, with the greatest effect observed in the apical and middle thirds. The control group's consistent mean of 0.0000 across all sections serves as a crucial baseline, highlighting the extent of dentinal thickness reduction due to the treatments. The significant P-values across all sections reinforce the substantial effect of the treatments on dentinal thickness, with a clear differentiation in the efficacy and impact of the treatment modalities represented by Groups A, B, and C. This analysis underscores the importance of treatment selection in preserving dentinal structure and integrity during endodontic procedures.

**Figure 3:**
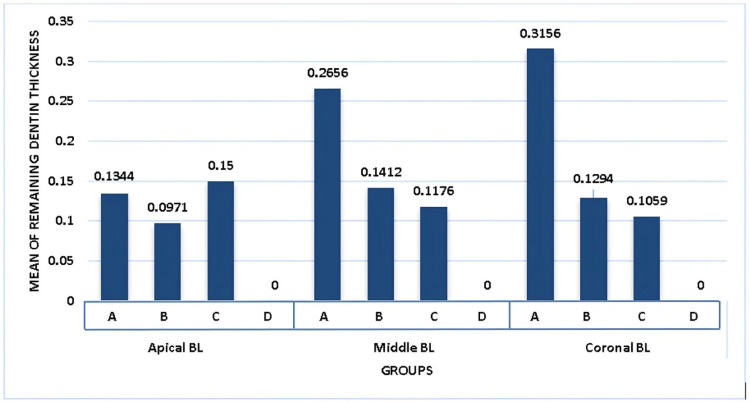
Graphical representation of intergroup comparison for mean buccolingual direction at coronal, middle and apical thirds of each tooth X-axis represents groups of different file systems. Y-axis represents the mean value of remaining dentin thickness.

**Figure 4:**
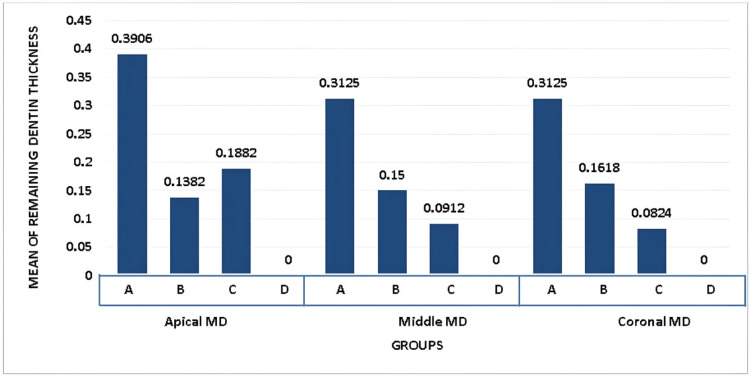
Graphical representation of intergroup comparison for mean mesiodistal direction at coronal, middle and apical thirds of each tooth X-axis represents groups of different file systems. Y-axis represents the mean value of remaining dentin thickness.

Figures 3, 4 illustrate the mean values of removed dentin at each level in both BL and MD, correspondingly. The Protaper universal system had the highest mean value at two mm in the MD. In group three, the WaveOne Gold file system indicated a low mean value of removed dentin in both directions, MD and BL, at 5 mm and 8 mm, respectively. The mean value at 2 mm in both directions was lowest in group two, the Protaper Gold system.

WaveOne Gold demonstrates significantly greater efficacy in preserving dentin thickness in the BL and MDs at the coronal as well as middle thirds contrasted to the apical third (p=0.001). Protaper Gold demonstrated statistical significance (p<0.05) in both the BL and MDs for preserving dentin thickness at the apical third in comparison to the coronal and middle third.

## Discussion

The three key components of endodontic therapy success are efficient disinfection, complete canal debridement, and canal obturation [13]. Biomechanical preparation is, therefore, the most crucial phase of endodontic therapy and is considered the most important stage with a predicted success factor when done right. Adequate taper across the root canal space is necessary for efficient irrigation and obturation; however, excessive expansion unnecessarily damages the root structure [14]. For this reason, selecting the right instrument to use instrumentation is essential to the success of root canal therapy.

Residual dentine thickness was measured using CBCT in the present study, which provides a useful non-destructive method for measuring the remaining dentin thickness of roots before and after shaping. CBCT provides cross-sectional and three-dimensional pictures that are highly measurable and accurate [15]. Other studies used the benefits of CBCT in measuring dentin thickness [16].

An investigation by Moore, Walter, & Parashos in 2009 compared hand instrumentation with rotational NiTi instruments. The conclusion indicated that the former method facilitated the canal preparation to wider apical diameters with less iatrogenic mistakes compared to hand instrumentation while being less conservative about apical root dentin [17]. According to a study conducted in 2001 by Raiden et al., radiographs frequently provide the impression that dentin is thicker than it actually is. CBCT was utilized in the current investigation because it made it possible to observe the root canal space in three dimensions as well as to estimate the amount of dentin removed before along with after instrumentation utilizing transverse, axial, as well as tangent assessments [18]. Three root canal system sections-the apical, middle, as well as coronal thirds, at 2 mm, 5 mm, and 8 mm correspondingly, where iatrogenic mishaps are most likely to occur [19].

In the present study, at 2 mm, the Protaper Gold file system revealed less dentin removal compared to other groups in both the BL and MDs and the difference was significant. This might be because of its better metallurgy and design. However, because of the ProTaper instruments' convex triangular cross-section, which lessens the area of contact between the file and dentin, the rotational method performed better at 2 mm in the current investigation. The design's inherent improved cutting efficiency has been enhanced by carefully adjusting the pitch and helix angle to stop the instruments from unintentionally screwing into the canal. The ProTaper system works through an active cut at a negative rake angle that significantly improves the overall effectiveness of the system while minimizing torsional stress [20]. According to the manufacturer, it is more resistant to fatigue than Protaper Universal. The design reduces the possibility of file binding inside the canal while enabling effective cutting action and debris removal. Its progressive tapering design allows for smooth and controlled shaping while maintaining the root canal's natural anatomy [21,22].

Waveone Gold removed the least dentin in the middle and coronal part in both mesiodistal as well as BL directions while Protaper Universal showed the highest followed by Protaper Gold. The most probable cause for the increased dentin removal at the coronal level is because the apical portion of Protaper has less taper than the coronal level.

In contrast to circular motion, the NiTi rotary instrument's reciprocating motion was just introduced (WaveOne, 2011) (DentsplyMaillefer, Ballaigues, Switzerland) to lessen the effects of cycle fatigue. Consequently, the single-file shaping approach has lately been suggested as a way to streamline instrumentation processes and reduce the chance of cross-contamination.

A single-file, single-use method for reciprocating motion, WaveOne Gold had been created based on the M-wire WaveOne instrument. Every file features a cross-sectional design with an alternating offset parallelogram form as well as a semi-active guiding tip. Another key distinctive design feature is that all files have a fixed taper from D1 to D3 but gradually decrease the percentage-tapered design from D4 to D16, contributing to dentin preservation. Its zone of interaction is bounded by an offset parallelogram-shaped cross-section. In addition, it has a reverse helix, semi-active and modified guiding tip, gold wire, new supermetal technology, and unusual, uneven reciprocating action in both directions [23].

Limoeiro et al., Venino et al., and Rubio et al. have reported on the residual dentin thickness after instrumentation; however, limited studies have investigated the remaining dentin thickness in two dimensions: mesiodistal as well as BL [24-26]. Therefore, the goal of the current investigation was to measure the amount of dentin extracted in the BL and MDs.

It was recommended that Protaper Gold and WaveOne Gold be applied with a brushing motion during the outstroke [27]. The larger, more potent, and more effective blades can passively flow farther into the canal when there is greater lateral space created by a brush-cutting action [28].

Another in vitro study assessed the residual dentin thickness of teeth with three rotating instruments: Protaper Universal, Protaper NEXT, and One Shape, conducted by Ramanathan et al. They concluded that Protaper Universal and Protaper NEXT should be used very conservatively because, compared with One shape, they showed significantly higher root dentin thinning [29].

A similar investigation on the comparison of Protaper as well as WaveOne file systems utilizing CBCT was carried out by Puri et al. in 2016. They found the Protaper file system showed a higher value of residual dentin thickness than the reciprocating file system; however, the difference was not statistically significant [30].

Clinical implications

It could help clinicians choose the best rotary system for preserving dentin thickness in single-rooted teeth. Identifying which rotary systems remove less dentin can aid in selecting instruments that prioritize structural integrity, especially in cases where dentin thickness is already compromised. Understanding which rotary systems are more conservative in dentin removal enables clinicians to minimize over-instrumentation, reducing the risk of procedural errors such as root perforation, excessive thinning, and weakening of the root structure. This is especially relevant for curved canals, apical third regions, or teeth with pre-existing thin dentin.

Strengths

Multiple rotary systems allows for direct comparison of their effects on dentin thickness. This design can provide insights into which system preserves more dentin, aiding clinicians in selecting appropriate tools based on RDT conservation, multiple systems can also help identify specific design elements (e.g., taper, tip size, cutting efficiency) that may impact dentin removal. Such findings could guide future advancements in rotary file design with improved RDT conservation. CBCT offers high-resolution, 3D imaging, providing precise measurements of RDT from multiple angles. This detailed view allows researchers to capture accurate pre- and post-instrumentation dentin thickness, improving the reliability of measurements compared to traditional 2D radiographs.

Limitations

Single-rooted teeth alone limit generalizability to multi-rooted teeth, where canal curvature and root morphology are more complex and may affect the dentin thickness differently with the same systems. Studies done on extracted teeth (in vitro) lack the biological variables present in a living tooth, such as periodontal ligament forces, hydration, and bone support, which can influence how dentin responds to instrumentation.

Comparable in vivo investigations with bigger sample sizes must corroborate the findings of this in vitro investigation. To minimize dentin removal during instrumentation and lower the incidence of endodontic failures, NiTi endodontic instrument design features and metallurgy require constant improvement. Considering all of the study's limitations, it was discovered that while some rotary files' design elements have a negative influence on the thickness of the remaining dentin; overall, these negative impacts are outweighed by the advantages of using these rotary tools. In light of the above results, WaveOne file systems are advised as an alternative to the widely utilized hand and rotary file systems.

## Conclusions

The present study aimed to compare the thickness of dentin removed after teeth were biomechanically prepared using different working techniques by rotary file systems. Results indicated that certain file systems demonstrated varying values of dentin removal at the apical, middle, as well as coronal levels. In both directions, BL and MD, the mean value of removed dentin at the apical area was the lowest for Protaper Gold. Compared to other file systems, the Waveone Gold file system reflected the lowest mean value of removed dentin at both the coronal and middle levels. This is shown in each working file system so far, depicting different levels of cutting efficiency at three different lengths: 2 mm, 5 mm, and 8 mm. Generally, however, Waveone Gold outperformed other file systems.

## References

[1] Shaheen NA, Farag AM, Alhadainy HA, Darrag AM (2013). Fracture resistance of endodontically treated roots using different preparation-obturation combinations. Tanta Dental J.

[2] Berutti E, Chiandussi G, Paolino DS, Scotti N, Cantatore G, Castellucci A, Pasqualini D (2012). Canal shaping with WaveOne primary reciprocating files and ProTaper system: a comparative study. J Endod.

[3] Bier CA, Shemesh H, Tanomaru-Filho M, Wesselink PR, Wu MK (2009). The ability of different nickel-titanium rotary instruments to induce dentinal damage during canal preparation. J Endod.

[4] Shahriari S, Abedi H, Hashemi M, Jalalzadeh SM (2009). Comparison of removed dentin thickness with hand and rotary instruments. Iran Endod J.

[5] Tambe VH, Nagmode PS, Abraham S, Patait M, Lahoti PV, Jaju N (2014). Comparison of canal transportation and centering ability of rotary protaper, one shape system and wave one system using cone beam computed tomography: an in vitro study. J Conserv Dent.

[6] Unb B, Roy M, Utpal K, Das C, Dutta K (2017). Comparison of apical transportation and centering ability of ProTaper next, HyFlex cm and twisted files by using cone beam computed tomography. J Med Dental Sci Res.

[7] Caputo AA, Standlee JP (1976). Pins and posts—why, when and how. Dental Clin North Am.

[8] Lim SS, Stock CJ (1987). The risk of perforation in the curved canal: anticurvature filing compared with the stepback technique. Int Endod J.

[9] Katz A, Tamse A (2003). A combined radiographic and computerized scanning method to evaluate remaining dentine thickness in mandibular incisors after various intracanal procedures. Int Endodont J.

[10] Deka A, Bhuyan AC, Bhuyan D (2015). A comparative evaluation of root canal area increase using three different nickel-titanium rotary systems: an ex vivo cone-beam computed tomographic analysis. Contemp Clin Dent.

[11] Ansari I, Maria R (2012). Managing curved canals. Contemp Clin Dent.

[12] Gergi R, Arbab-Chirani R, Osta N, Naaman A (2014). Micro-computed tomographic evaluation of canal transportation instrumented by different kinematics rotary nickel-titanium instruments. J Endod.

[13] Pathak S (2016). In vitro comparison of K-file, Mtwo, and WaveOne in cleaning efficacy and instrumentation time in primary molars. CHRISMED J Health Resources.

[14] Zandbiglari T, Davids H, Schäfer E (2006). Influence of instrument taper on the resistance to fracture of endodontically treated roots. Oral Surg Oral Med Oral Pathol Oral Radiol Endod.

[15] Alrahabi M, Alkady A (2017). Comparison of root canal apical transportation associated with wave one, ProTaper next, TF, and Oneshape nickel-titanium instruments in curved canals of extracted teeth: a radiographic evaluation. Saudi J Dental Res.

[16] Nagaraja S, Sreenivasa Murthy BV (2010). CT evaluation of canal preparation using rotary and hand NI-TI instruments: an in vitro study. J Conserv Dent.

[17] Moore J, Fitz-Walter P, Parashos P (2009). A micro-computed tomographic evaluation of apical root canal preparation using three instrumentation techniques. Int Endod J.

[18] Sousa K, Andrade-Junior CV, Silva JM, Duarte MA, De-Deus G, Silva EJ (2015). Comparison of the effects of triplegates and Gates-Glidden burs on cervical dentin thickness and root canal area by using cone beam computed tomography. J Appl Oral Sci.

[19] Elnaghy AM, Elsaka SE (2014). Evaluation of root canal transportation, centering ratio, and remaining dentin thickness associated with ProTaper next instruments with and without glide path. J Endod.

[20] Clauder T, Baumann MA (2004). ProTaper NT system. Dent Clin North Am.

[21] Yalniz H, Koohnavard M, Oncu A, Celikten B, Orhan AI, Orhan K (2021). Comparative evaluation of dentin volume removal and centralization of the root canal after shaping with the ProTaper Universal, ProTaper gold, and one-curve instruments using micro-CT. J Dent Res Dent Clin Dent Prospects.

[22] Hieawy A, Haapasalo M, Zhou H, Wang ZJ, Shen Y (2015). Phase transformation behavior and resistance to bending and cyclic fatigue of ProTaper gold and ProTaper Universal Instruments. J Endod.

[23] Ruddle C (2016). Advanced Endodontics. https://www.endoruddle.com/tc2pdfs/show/201/WaveOneGold_Jan2016.pdf.

[24] Venino PM, Citterio CL, Pellegatta A, Ciccarelli M, Maddalone M (2017). A micro-computed tomography evaluation of the shaping ability of two nickel-titanium instruments, HyFlex EDM and ProTaper next. J Endod.

[25] da Silva Limoeiro AG, Dos Santos AH, De Martin AS (2016). Micro-computed tomographic evaluation of 2 nickel-titanium instrument systems in shaping root canals. J Endod.

[26] Rubio J, Zarzosa JI, Pallarés A (2017). Comparison of shaping ability of 10 rotary and reciprocating systems: an in vitro study with AutoCAD. Acta Stomatol Croat.

[27] Ruddle C: S HAPING COMPLEX CANALS CLINICAL STRATEGY AND TECHNIQUE (2014). Shaping complex canals clinical strategy and technique. https://www.endoruddle.com/tc2pdfs/show/231/ProTaperGOLD_Nov2014.pdf.

[28] Ruddle CJ (2005). The ProTaper technique. Endodont Top.

[29] Ramanathan S, Solete P (2015). Cone-beam computed tomography evaluation of root canal preparation using various rotary instruments: an in vitro study. J Contemp Dent Pract.

[30] Puri P, Mishra A (2022). Comparative evaluation between two Niti rotary files using CBCT. Int Healthcare Res J.

